# Early Prostatectomy

**Published:** 1908-09

**Authors:** 


### ABSTRACTS.

---

### Early Prostatectomy.

Miles F. Porter, Fort Wayne, Ind. (*Journal A. M. A.*, May 23), pleads for early prostatectomy in cases of prostatic hypertrophy requiring operation. In the analysis of 485 recorded cases, occurring in the surgical practice of thirteen different operators, he finds a total death rate of less than 7 per cent., while the death rate of the operation itself is less than 2 per cent., and the death rate from conditions secondary to and caused by the enlarged prostate is 3.5 per cent. In other words, half of all the deaths following prostatectomy are due to the diseased condition and outnumber those from the operation *per se* two to one. This means that the death rate in hypertrophy of the prostate treated without operation is about 4 per cent., while timely prostatectomy will yield a death rate of 2 per cent. or less. If there is any error here, he says, it is in attributing too many deaths to the operation and charging too few to pathologic conditions. Turning next to the post-operative morbidity, he finds urinary incontinence, stricture and fistula very exceptional results: Fuller, in 400 cases, has had but one permanent urinary dribbling. Barling, Hartmann, McGill, Lilienthal and Freyer are of the opinion that control of the bladder is not interfered with by prostatectomy, but, on the contrary, is often restored. For obvious reasons, exact statistics as to the effect of prostatectomy on the sexual functions are difficult to obtain, and Porter thought the best way to obtain a just estimate would be to get the statement from a number of operators of experience as to their impressions derived from their patient's testimony on this point. The answers to his inquiries, which are given in his article would seem to indicate that the results have been generally satisfactory to the patients. Of course prostatectomy will not restore senile incapacity. His personal experience is limited to 25 cases. There were three deaths due to preëxisting septic conditions for which operative relief was demanded. There were no postoperative sequelæ, and he knows of no operation that gives more general and complete satisfaction to the sufferers. That malignant disease is present in a much larger proportion of cases than has been supposed, is proved by the experience of Young, the Mayos, Vander Veer, Monro, Bottomley and others, and that a benign hypertrophy may become malignant, appears, from the evidence, a well-nigh proved fact. Porter concludes, from all the data, that since catheter treatment is unsurgical and unsafe, prostatectomy, performed before secondary changes have occurred, is the best treatment for enlarged prostate.

